# Comparison of keratometric measurements between color light-emitting diode topography and Scheimpflug camera

**DOI:** 10.1186/s12886-019-1106-1

**Published:** 2019-04-26

**Authors:** Xian-Hua Cui, Young-Sik Yoo, Youngju An, Choun-Ki Joo

**Affiliations:** 1 Department of Ophthalmology and Visual Science, College of Medicine, Seoul St. Mary’s Hospital, The Catholic University of Korea, 222 Banpo-daero, Seocho-gu, Seoul, 06591 South Korea; 2 Department of Ophthalmology, Yanbian University Hospital, #1327 Juzi St, Yanji, 133000 Jilin Province People’s Republic of China; 3 Department of Ophthalmology, Samsung Medical Center, Sungkyunkwan University School of Medicine, 81 Ilwon-ro, Gangnam-gu, Seoul, 06351 South Korea; 4 Department of Optometry, Baekseok Culture University, 58, Munam-ro, Dongnam-gu, Cheonan-si, Chungcheongnam-do South Korea

**Keywords:** Astigmatism, Cornea, Ocular refraction

## Abstract

****Background**:**

To determine the agreement of measurements between color light-emitting diode corneal topography (Cassini) and Scheimpflug camera keratometry (Pentacam HR).

**Methods:**

The current retrospective study investigated 117 right eyes of 117 healthy patients before cataract surgery from June 2017 to July 2017. Steep K, flat K, mean K, astigmatism, and axis for both anterior and posterior corneal surface were measured using the two devices. The measured values were converted into J vectors such as J0 and J45. The mean difference for those measurement values were compared between the two instruments, and the agreement was evaluated using the Bland-Altman plot I.

**Results:**

There were statistically significant differences in mean K (44.21D [43.34 to 45.34] and 44.30D [43.30 to 45.10] by Cassini and Pentacam [*P* = 0.004]) and astigmatism (0.90D [0.58 to 1.30] and 0.70D [0.40 to 1.30] by Cassini and Pentacam [*P* = 0.002]) on the anterior corneal surface and flat K (− 6.21D [− 6.39 to − 6.07] and − 6.30D [− 6.5 to − 6.10] by Cassini and Pentacam [*P* < 0.001]), mean K (− 6.39D [− 6.54 to − 6.25] and − 6.40D [− 6.60 to − 6.30] by Cassini and Pentacam [*P* = 0.019]), and astigmatism (0.33D [0.22 to 0.47] and 0.30D [0.15 to 0.40] by Cassini and Pentacam [*P* = 0.002]) on the posterior corneal surface. The mean difference (= Cassini – Pentacam) with 95% limit of agreement for mean K and astigmatism of the anterior corneal surface were 0.082D (− 0.60 to 0.76) and 0.11D (− 0.73 to 0.95) for measurements obtained by the two instruments, respectively. Regarding keratometric values from the posterior corneal surface, the mean differences for flat K, mean K, and astigmatism were − 0.081D (− 0.42 to 0.26), − 0.030D (− 0.32 to 0.26), and 0.067D (− 0.33 to 0.46), respectively. Intraclass correlation coefficients for steep K, flat K, mean K, and vector J0 were higher than 0.9 in the anterior cornea. Positive correlation in steep K, flat K, mean K, astigmatism, and J0 was found between two devices in both anterior and posterior cornea (*P* < 0.001).

**Conclusions:**

Corneal refractive power and astigmatism tend to be higher when measured using Cassini than Pentacam HR in both anterior and posterior cornea. The two different devices might not be used interchangeably.

**Trial registration:**

Retrospectively registered. Registration number: KC17**RESI**0439.

## Background

In cataract surgery, refractive power of the cornea plays an important role in the calculation of power in artificial intraocular lens (IOL) insertion. Accurate measurement of the corneal refractive power and astigmatism are crucial factors in intraoperative correction of astigmatism as well as for improvement of the patient’s vision and satisfaction with treatment [1, 2]. Both the magnitude and axis of the anterior and posterior cornea determine the overall corneal astigmatism [3].

Among devices used for anterior segment biometry, Pentacam HR (software version 6.07r12, Oculus, Wetzlar, Germany), a type of rotational Scheimpflug camera, and the recently developed Cassini (software version 2.4.0, i-optics, The Hague, Netherlands) [4] which uses the color-LED method, can measure the curvature of not only the anterior but also the posterior cornea. Recently, studies that emphasize the importance of posterior corneal astigmatism in cataract surgery for the minimization of postoperative remnant astigmatism have increased [5–7]. The clinical applicability of these devices is increasing the accuracy for the actual corneal astigmatism measurement using the posterior corneal astigmatism, especially in toric IOL implantation [3, 8]. Pentacam HR analyzes the tomographic images obtained from a single rotating camera, whereas Cassini is a new topographer that uses a multicolor (red, yellow, and green) spot pattern, which analyses the specular reflection using 679 Light-emitting diode (LED) spots superimposed on the cornea. Pentacam HR corrects errors caused by eye movements and plentiful data are present in the literature regarding the good or excellent repeatability and reproducibility of data measured using Pentacam HR. However, there are some reports that Pentacam HR has low repeatability due to eye movement because of its long measurement time [9], whereas Cassini reconstructs the specular reflections from 679 color-LEDs by a point-to-point method, thereby instantly analyzing the shapes of the anterior and posterior cornea [5].

This study aims to comparatively analyze the refractive power, astigmatism, and axis of the anterior and posterior cornea and assess whether the two devices can be used interchangeably.

## Methods

A retrospective study was conducted on 117 eyes of 117 patients undergoing normal cataract surgery in our center’s Ophthalmology Department from June 7, 2017 to July 27, 2017. The stage of cataract for them ranged from 1 to 4 by Pentacam nucleus staging system. Right eye was selected from each subject. The study was conducted in adherence to the Declaration of Helsinki as well as under the approval of the Medical Research Ethics Board of the Catholic University of Korea (IRB approval number: KC17**RESI**0439). Eyes with preoperative pathologies that could affect corneal astigmatism, such as pterygium, severe corneal turbidity, corneal pathology due to severe dry eye, and diseases of the eyelid, orbit, and conjunctiva were excluded from the analysis.

Two investigators measured the curvature of the anterior and posterior cornea, as well as astigmatism, prior to surgery using color light-emitting diode corneal topography (Cassini; i-Optics, Hague, Netherlands) and Scheimpflug camera keratometry (Pentacam HR; Oculus, Wetzlar, Germany) in that order, at an interval of under 10 min. Keratometry of their eyes was performed before applying any kind of eye drops. We performed the procedure after guiding the subject to fix their chin and forehead on the equipment’s test bed and to observe the internal fixation target in the test equipment while sitting in front of the test equipment. In order to minimize errors in measurement due to changes in the tear layer, patients were encouraged to blink sufficiently before each measurement. The same investigator took three measurements, and the average of the three values was used in subsequent analyses to compare each test result. Both Cassini and Pentacam HR provided qualitative information, and only those with “OK” or “measurement successful” results were included in the analysis.

Cassini reconstructs the specular reflections from 679 color-LEDs by a point-to-point method, thereby instantly analyzing and measuring the shapes of the anterior and posterior cornea, the corneal curvature, lower-order aberration, and higher-order aberration [3, 10]. The Pentacam HR hardware comprises a single rotating camera. It obtains tomographic images of the anterior segment by means of 2-s scans through a 450-nm blue light-emitting diode using the same principles as that of a 360° rotating Scheimpflug camera [11]. However, keratometry using the Scheimpflug method has a relatively long measurement time and is sensitive to eye movement.

Cassini and Pentacam HR were both used to measure the steep K, flat K, mean K, astigmatism, and axis of the anterior and posterior sides of a central 3-mm corneal area (simulated keratometry). J0 and J45, which are Jackson cross-cylinder (JCC) values, were used for the analysis of astigmatism through power vector analysis [12]. The magnitude and axis were measured three times and expressed as J0 and J45, respectively. The average values for both J0 and J 45 were calculated and then converted back to magnitude and axis. The formula to obtain the Jackson cross-cylinder is as follows:

$$ \mathrm{J}0=-\mathrm{C}/2\times \cos 2\uptheta $$

$$ \mathrm{J}45=-\mathrm{C}/2\times \sin 2\uptheta $$

$$ \left(\mathrm{C};\mathrm{negative}\ \mathrm{astigmatism}=\mathrm{flat}\ \mathrm{K}-\mathrm{steep}\ \mathrm{K};\uptheta =\mathrm{flat}\ \mathrm{meridian}\right) $$

SPSS software (ver. 18.0; SPSS Inc., Chicago, IL) and R software (ver. 3.5.2; R foundation for Statistical Computing, Vienna, Austria) were used for statistical analyses. The paired t-test was used to compare the average measured values between the two devices. Intraclass correlation coefficient (ICC) was calculated to evaluate the degree of agreement between the two devices. Pearson correlation was used to obtain the correlation coefficient between the measurement methods and the scatter plots were produced using the LOWESS curve. The agreement between the values measured using the two devices was analyzed by the Bland-Altman plot and is expressed as 95% limits of agreement. *P* values of less than 0.05 were deemed statistically significant.

## Results

Mean patient age was 65.1 ± 11.5 years (range 22–93). Thirty-four subjects (29%) were men, and 57 eyes (49%) were right eyes. The median astigmatism values for both anterior, posterior, and total cornea were larger in Cassini than in Pentacam HR (Table 1).

**Table 1:** Overview of astigmatism magnitude

Device	Anterior Surface	Posterior Surface	Total Cornea
	Median [IQR] (Range) (D)	Median [IQR] (Range) (D)	Median [IQR] (Range) (D)
Cassini	0.90 [0.58–1.30] (0.09–3.32)	0.33 [0.22–0.47] (0.04–0.89)	0.72 [0.40–1.20] (0.04–3.02)
Pentacam HR	0.70 [0.40–1.30] (0.10–3.50)	0.30 [0.15–0.40] (0.00–0.80)	0.60 [0.34–1.18] (0.10–3.20)

*D* diopters, *IQR* interquartile range, *N* number of cases

The difference (= Cassini - Pentacam HR) in average values of the anterior cornea was 0.08D (− 1.30 to 1.50, *P* = 0.057) for steep K, 0.01D (− 1.10 to 0.80, *P* = 0.692) for flat K, 0.08D (− 0.89 to 1.02, *P* = 0.011) for mean K, 3.88° (− 174.9 to 174.3, *P* = 0.485) for axis, − 0.04D (− 0.74 to 0.53, *P* = 0.045) for vector J0, 0.00D (− 0.56 to 0.53, *P* = 0.845) for J45, and 0.11D (− 1.41 to 1.44, *P* = 0.004) for astigmatism (Table 2). In the anterior cornea, the mean K, vector J0, and astigmatism showed statistically significant differences. The difference in average values measured using the two devices in the posterior cornea was 0.00D (− 0.42 to 0.40, *P* = 0.791) for steep K, 0.08D (− 0.41 to 0.51, *P* = 0.000) for flat K, 0.03D (− 0.45 to 0.37, *P* = 0.033) for mean K, 4.25° (− 17.2 to 177.4°, *P* = 0.639) for axis, − 0.04D (− 0.35 to 0.24, *P* = 0.002) for vector J0, − 0.01D (− 0.39 to 0.36, *P* = 0.493) for J45, and 0.07D (− 0.41 to 0.76, *P* = 0.001) for astigmatism. In the posterior cornea, flat K, mean K, vector J0, and astigmatism showed statistically significant differences.

**Table 2:** Comparison of corneal refractive power (K) measured by Cassini and Pentacam HR

	Anterior corneal surface				Posterior corneal surface			
	Cassini	Pentacam HR	*P*-value*	ICC	Cassini	Pentacam HR	*P*-value*	ICC
Steep K	44.80 (41.60–48.80)	44.72 (41.30–48.40)	0.057	0.974	6.58(5.97–7.13)	6.58(5.80–7.20)	0.791	0.876
Flat K	43.82 (41.22–47.07)	43.81 (41.10–46.80)	0.692	0.981	6.23(5.64–6.90)	6.31(5.60–6.90)	0.000^**^	0.859
Mean K	44.34 (41.46–47.84)	44.25 (41.20–47.40)	0.011^**^	0.983	6.4(5.83–6.95)	6.43(5.70–7.00)	0.033^**^	0.892
Astigmatism	1.03 (0.09–3.32)	0.91 (0.10–3.50)	0.004^**^	0.886	0.35(0.04–0.89)	0.29(0.00–0.80)	0.001^**^	0.563
Axis	91.67 (0.00–179.00)	87.78 (1.10–178.10)	0.485	0.763	93.80(0.00–180.00)	89.55(0.20–179.70)	0.639	−0.422
J0	−0.02 (−1.33–1.36)	0.03 (− 0.84–1.67)	0.045^**^	0.945	0.09(− 0.28–0.39)	0.13 (− 0.12–0.40)	0.002^**^	0.591
J45	0.01 (− 0.96–0.98)	0.00 (− 0.79–0.52)	0.845	0.851	− 0.01(− 0.36–0.42)	0.00(− 0.13–0.25)	0.493	0.252

Values are presented as Mean (Range) (D)

*ICC* intra-class correlation coefficient

*Paired t-test

**Fig. 1:**
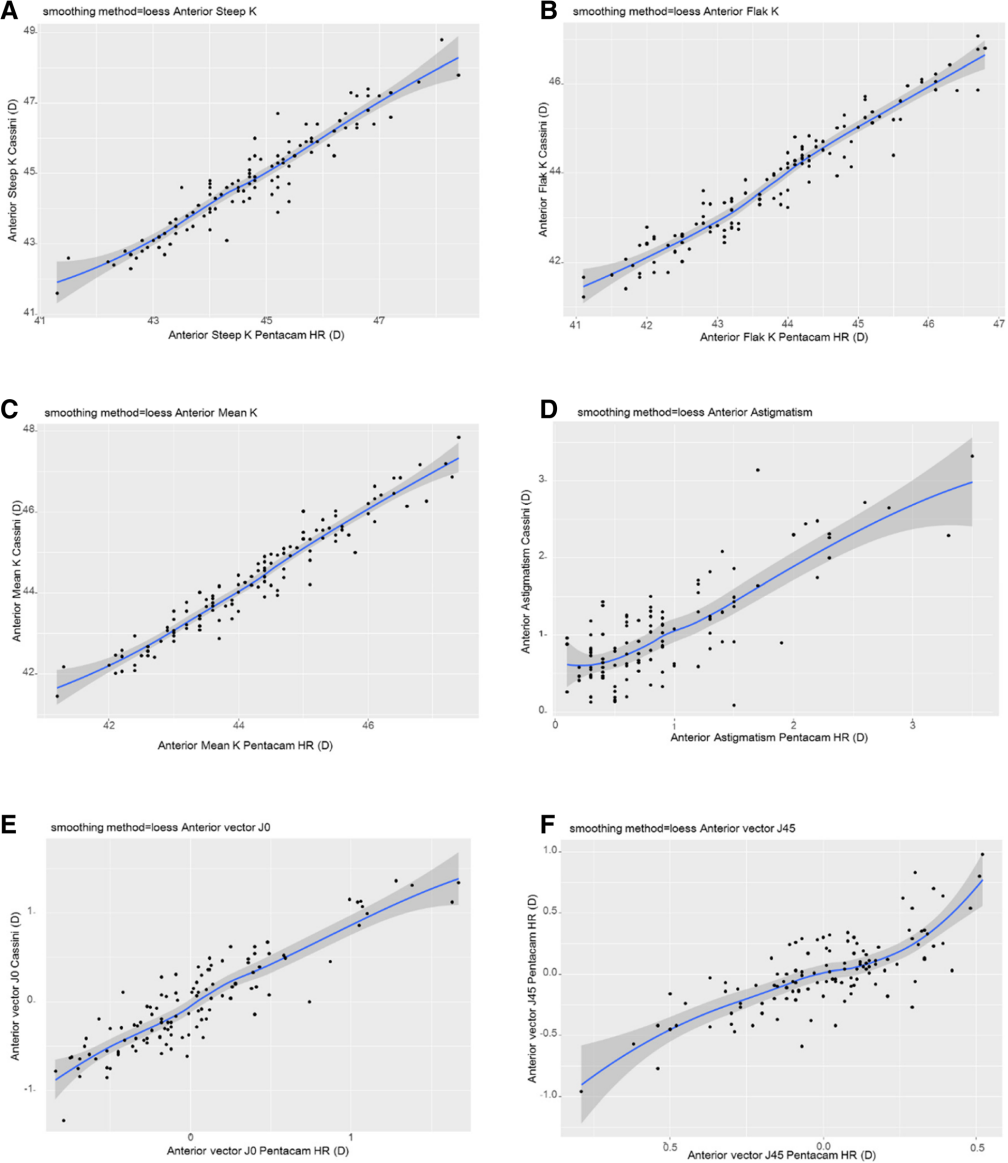
Pearson correlation of anterior segment parameters between Cassini and Pentacam HR. **a** Anterior Steep K, **b** Anterior Flat K, **c** Anterior Mean K, **d** Anterior Astigmatism, **e** Anterior vector J0, **f** Anterior vector J45. K=Keratometric diopter

**Fig. 2:**
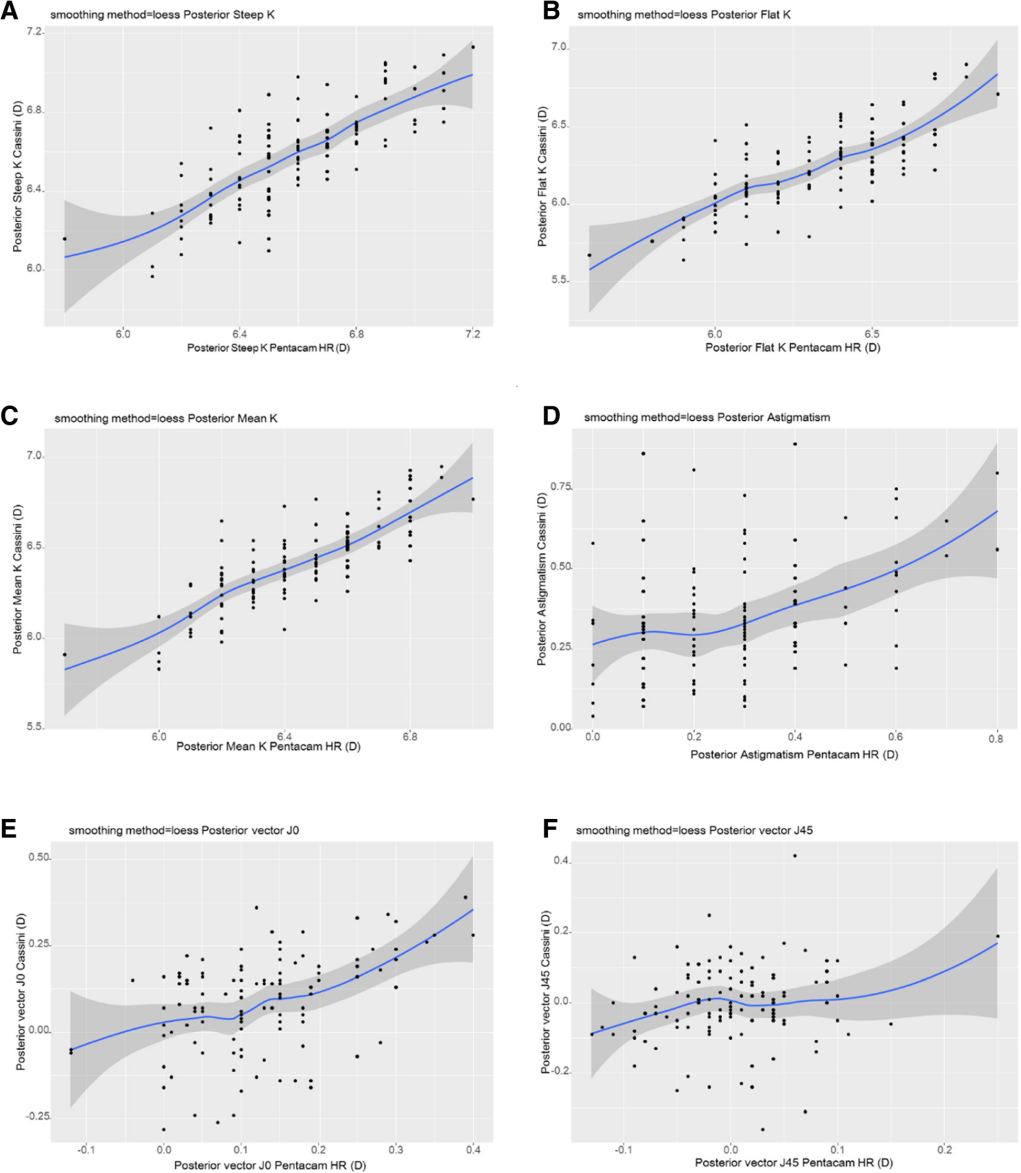
Pearson correlation of posterior segment parameters between Cassini and Pentacam HR. **a** Posterior Steep K, **b** Posterior Flat K, **c** Posterior Mean K, **d** Posterior Astigmatism, **e** Posterior vector J0, **f** Posterior vector J45. K=Keratometric diopter

ICC between the two devices were higher than 0.9 for steep K, flat K, mean K, and vector J0 in the anterior cornea and lower than 0.75 for astigmatism, axis, vector J0, and vector J45 in the posterior cornea. Pearson correlation showed significantly positive correlation in steep K, flat K, mean K, astigmatism, and vector J0 between the two devices: in the anterior cornea, r = 0.950, r = 0.962, r = 0.967, r = 0.796, r = 0.897, respectively (*P* < 0.001, Fig. 1); in the posterior cornea, r = 0.780, r = 0.753, r = 0.806, r = 0.392, r = 0.448, respectively (*P* < 0.001, Fig. 2). In the anterior cornea, J45 showed a significant positive correlation between the two devices (r = 0.757, *P* < 0.001); however, in the posterior cornea, J45 showed no significant correlation (r = 0.176, *P* = 0.058, Figs. 1 and 2).

**Fig. 3:**
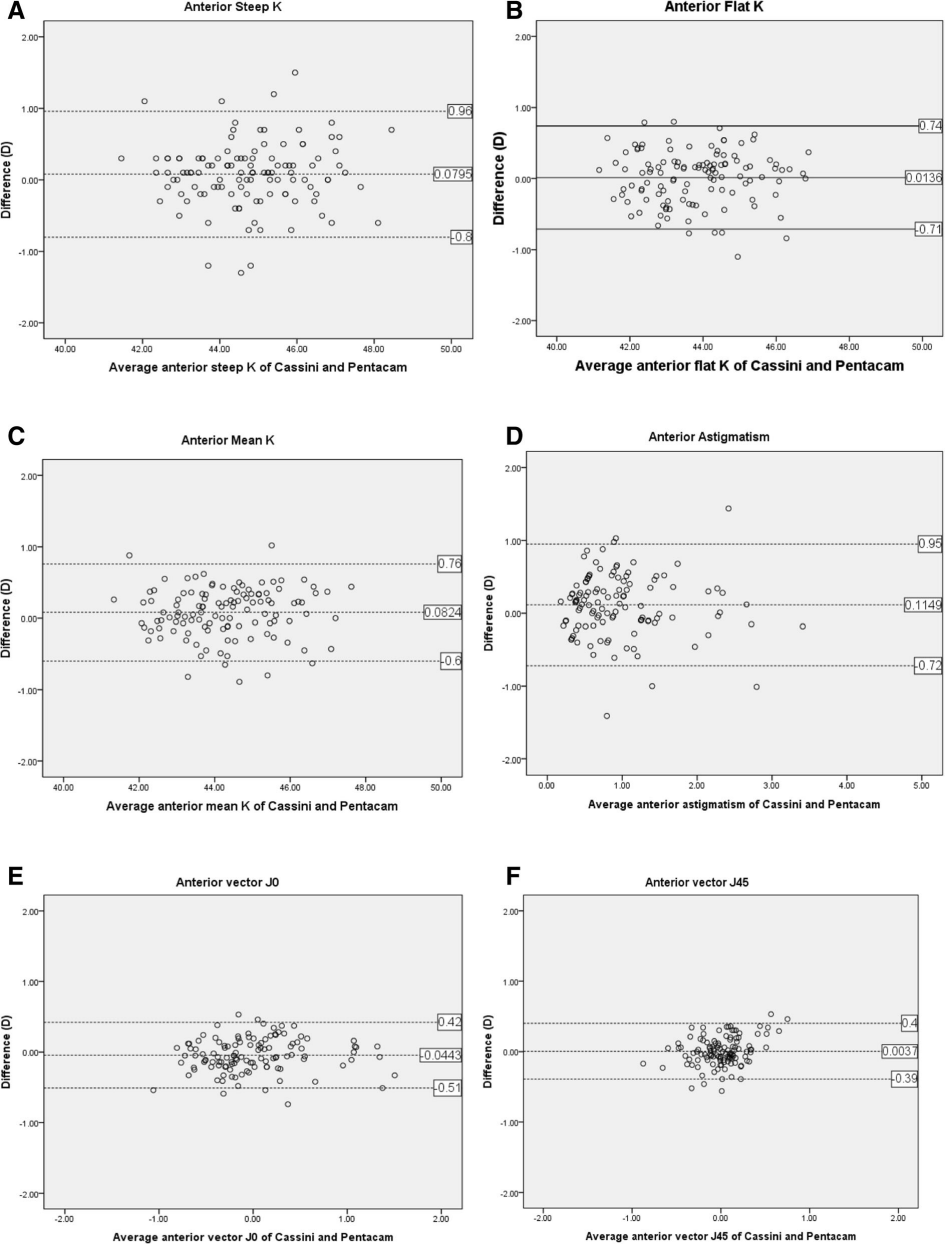
Bland-Altman plots of anterior segment parameters using Cassini and Pentacam HR. **a** Anterior Steep K, **b** Anterior Flat K, **c** Anterior Mean K, **d** Anterior Astigmatism, **e** Anterior vector J0, **f** Anterior vector J45. K=Keratometric diopter

**Fig. 4:**
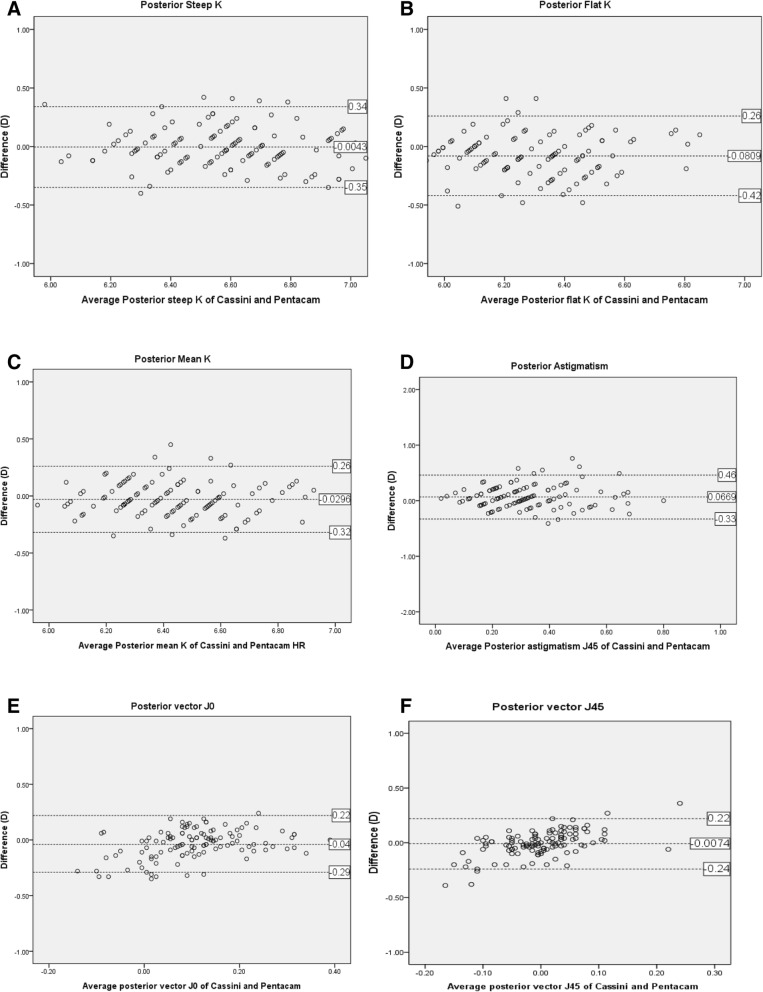
Bland-Altman plots of posterior segment parameters using Cassini and Pentacam HR. **a** Anterior Steep K, **b** Anterior Flat K, **c** Anterior Mean K, **d** Anterior Astigmatism, **e** Anterior vector J0, **f** Anterior vector J45. K=Keratometric diopter

The range of 95% limits of agreement in the anterior cornea was − 0.80 to 0.96D for steep K, − 0.71 to 0.74D for flat K, − 0.60 to 0.76D for mean K, − 0.72 to 0.95D for astigmatism, − 0.51 to 0.42D for J0, and − 0.39 to 0.40D for J45 (Fig. 3); and in the posterior cornea, − 0.34 to 0.35D for steep K, − 0.26 to 0.42D for flat K, − 0.26 to 0.32D for mean K, − 0.33 to 0.46D for astigmatism, − 0.29 to 0.22D for J0, and − 0.24 to 0.22D for J45 (Fig. 4). The amount of anticipated residual refractive astigmatism and axis did not differ between the two groups (Table 3). As a result, it is predicted that astigmatism of the toric IOL will not be affected by the two devices.

**Table 3:** Anticipated residual refractive astigmatism calculated by Alcon online toric intraocular lens calculator using keratometric values from Cassini and Pentacam HR

	Cassini	Pentacam HR	*P*-value*
Magnitude	0.91 ± 0.57	0.93 ± 0.66	0.965
Meridian	99 ± 69	85 ± 74	0.487

Values are presented as Mean ± SD

*Paired t-test

## Discussion

The observed corneal refractive power and astigmatism tended to be higher when using Cassini than when using Pentacam in the anterior and posterior cornea in the present study. The 95% limit of agreement was quite large for most variables measured, especially for the anterior corneal surface, comfortably extending beyond 0.5 D except for J0 and J45. The results for the posterior surface shared similar characteristics. This shows that the agreement was low from a clinical point of view.

Studies have reported that ignoring the posterior corneal values may result in erroneous measurement of overall astigmatism [5, 13]. In WTR astigmatism, ignoring the posterior astigmatism may cause overcorrection. In younger patients, in particular, slight WTR astigmatism must remain after correction, considering the tendency to progress to ATR astigmatism with age. Therefore, when inserting an IOL for the correction of astigmatism, care must be taken to consider posterior astigmatism and avoid overcorrection [14–16]. Additionally, one of the main limitations of Scheimpflug imaging is the low resolution and poor quality of anterior segment scans. In this regard, Cassini is known to produce better images with higher definition and to enable accurate measurement of the posterior cornea using multiple LED points. Although some researchers have evaluated its agreement with Pentacam HR, they focused on the measurement accuracy of total corneal astigmatism in toric IOL implantation or evaluated the agreement level in the relatively small sample size [3, 17]. The present study comparatively analyzed not only total corneal astigmatism but also refractive power, astigmatism, and the difference in astigmatic axes of the anterior and posterior cornea, and assessed whether the two devices can be used in a complementary manner.

Comparison of measurements using Cassini and Pentacam HR indicated significant differences in the average refractive power of the cornea and astigmatism in both the anterior and the posterior cornea. Pentacam HR reconstructs the images of the anterior segment into a 3-dimensional structure and allows for the visualization of the anterior and posterior cornea, as well as measurement of astigmatism in certain areas of the cornea. In a study on 493 eyes, Ho et al. [8] reported that corneal astigmatism measured by Pentacam HR showed a difference of 0.24 ± 0.16D in size and 7.4 ± 10.3° in axis compared to anterior astigmatism, possibly due to the presence of posterior corneal astigmatism. Klijn et al. [18] compared the refractive power of the cornea measured with Cassini, Keratron, Lenstar, and Pentacam HR and reported values of 43.42 ± 1.37D and 43.44 ± 1.46D using the Cassini and Pentacam HR, respectively, without significant difference (*P* = 0.64). In the present study, the average values measured in the anterior cornea by the Cassini and Pentacam HR was 44.34 ± 1.35D and 44.25 ± 1.34D, respectively and in the posterior cornea was 6.43 ± 0.23D and 6.43 ± 0.24D for mean K. The average corneal curvature and the average astigmatism size measured by the two devices showed small differences of maximum 0.43D and 0.54D in the anterior cornea and maximum 0.18D and 0.27D in the posterior cornea.

The two devices may not be used interchangeably. In the present study, the results of Pearson correlation analysis showed high correlation between the corneal refractive powers of the anterior and posterior cornea (r = 0.950 and r = 0.806, respectively); however, based on the 95% agreement limit of − 0.60~0.76D and − 0.26~0.32D in the anterior and posterior cornea, respectively. This might be caused that the two devices have different methods of measuring the corneal curvature and the accuracy of measurement or the calibration of each device affects the values. Though both devices measure the same 3-mm central corneal area, Pentacam HR requires a longer duration of time compared with Cassini; therefore, factors involved in patient cooperation, such as eye stability, compensatory saccadic movement, and consistent eye opening, would affect the test results.

The limitation of this study is that measurement error may have occurred, since two investigators were involved in the measurement and in addition, inter-reader agreement could not be determined. Moreover, the study was conducted only on normal corneas, and difference due to the innate characteristics of each device was not considered. In the present retrospective study, we calculated the required sample size using G Power for a power of 0.8. The number of required samples was 128. However, the number of samples was as low as 117 people. Additional studies that compensate for the above limitation and those with repeated measurements are required for results that are more accurate.

## Conclusions

In conclusion, both two devices are useful in measuring keratometry, but the two devices might be non-interchangeable.

## Abbreviations

ATR: Against-the-rule
DV: Difference Vector
ICC: Intraclass correlation coefficient
IOL: Intraocular Lens
IQR: Interquartile range
JCC: Jackson cross-cylinder
LED: Light-emitting diode
TCP: Total corneal power
WTR: Within-the-rule
